# A Four-Year Hospital Journey for a Lost and Migrating Appendicolith

**DOI:** 10.1155/2015/832434

**Published:** 2015-08-04

**Authors:** Daniel Schraffl, Herman Frima, P. Villiger

**Affiliations:** Department of Surgery, Cantonal Hospital Graubünden, Loëstrasse 170, 7000 Chur, Switzerland

## Abstract

We report a rare case of recurrent abscess formation, including being at a retroperitoneal site, due to a lost and migrating appendicolith. Over a four-year period and following an episode of perforated appendicitis, an otherwise healthy young man underwent two operations for abscess formation with eventual stone removal. Appendicoliths can pose a challenge during the diagnostic and therapeutic journey, and adequate attempts at removal need to be made to prevent avoidable complications. In patients suffering from recurrent flank pain and abscesses after appendectomy, the possibility of a lost or overlooked appendicolith should be considered.

## 1. Introduction

Here, we report a rare case of a retroperitoneal abscess arising due to a lost and migrating appendicolith. Our patient underwent two operations after initial appendectomy until the stone was eventually removed. This case highlights how a migrating appendicolith can pose a challenge during the diagnostic and therapeutic journey.

## 2. Material and Methods

A 32-year-old man presented to the Emergency Department in early 2010 with a three-day history of right abdominal pain, severe pain to pressure over McBurney's point, and clinical and biochemical signs of acute appendicitis. The patient underwent laparoscopic appendectomy on the same day. A perforated retrocaecal appendix was excised and acute appendicitis with abscess diagnosed. No appendicolith was seen intraoperatively, and the appendix was completely removed. The abdomen was flushed and a Jackson-Pratt drain inserted. After four days, the patient was discharged in good condition with a five-day course of antibiotics (ciprofloxacin/metronidazole).

**Figure 1:**
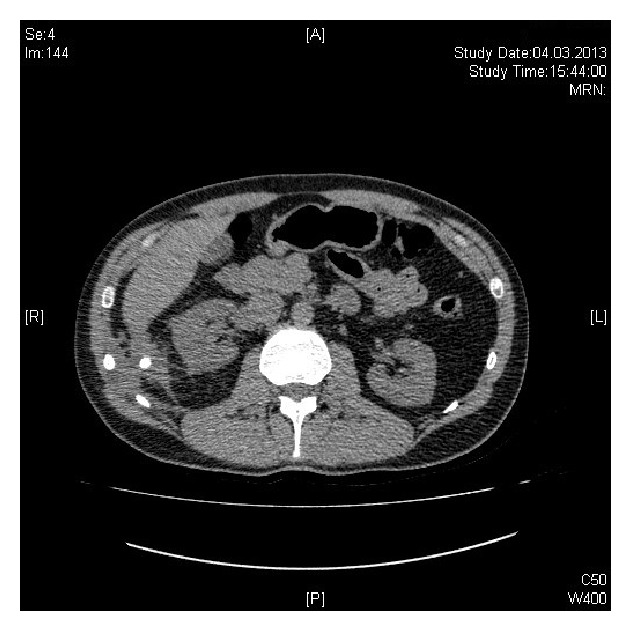


**Figure 2:**
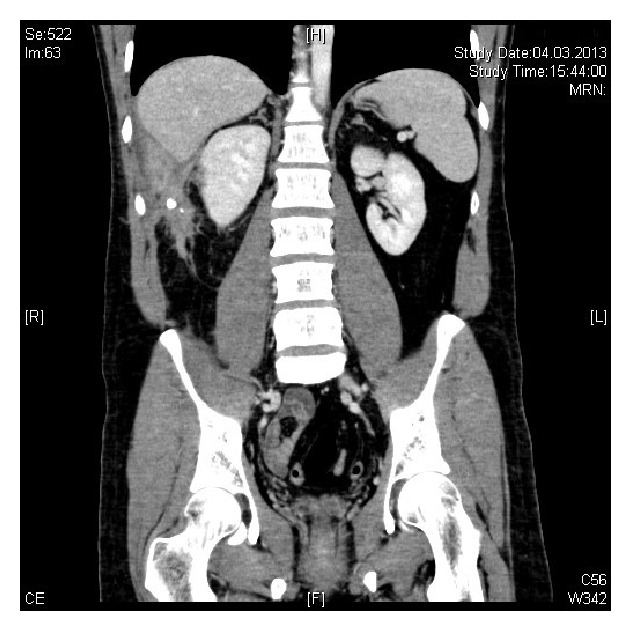


Three years later (2013), the patient was referred to Urology Department with recurrent right flank pain and a suspected kidney stone. No urinary calculus was visible on sonography. A CT scan with and without contrast was performed, and a right paracolic abscess containing an appendicolith was observed next to the ascending colon (Figures 1 and 2).

The right ascending colon required mobilisation at subsequent diagnostic laparoscopy. After incision of the abscess, pus was drained and lavage was performed. However, no appendicolith was found. Antibiotics (ciprofloxacin/metronidazole) were prescribed for fourteen days and the patient was discharged in good health after four days.

**Figure 3:**
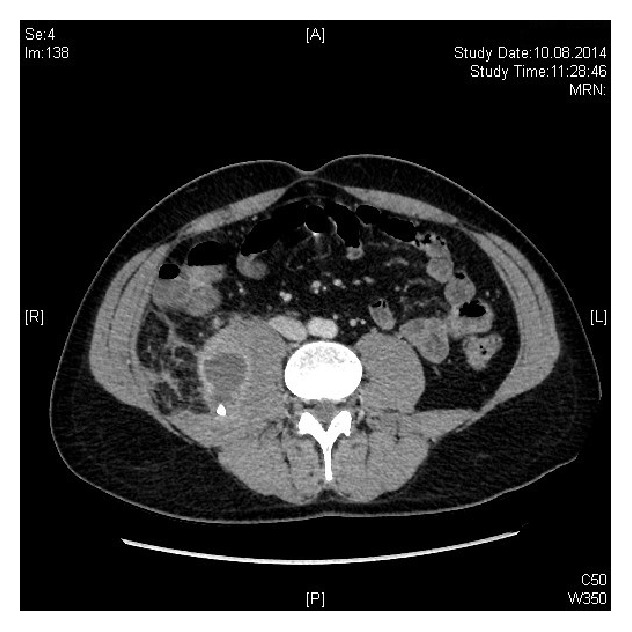


**Figure 4:**
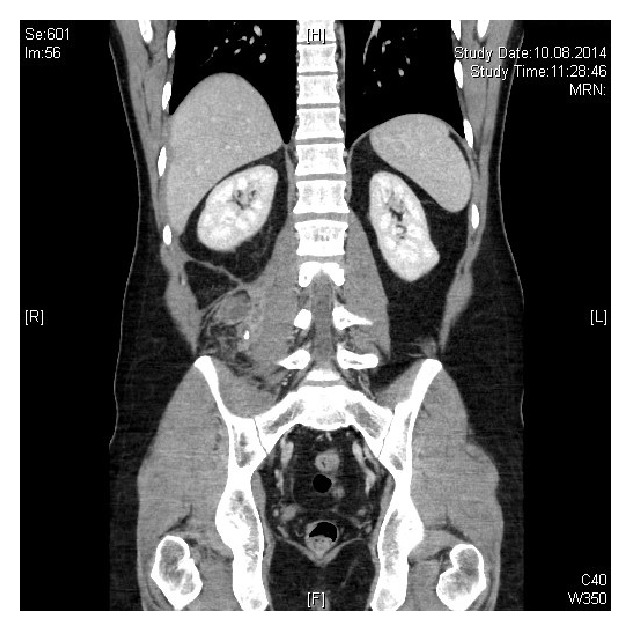


One year later (2014), the patient re-presented to the Emergency Department with a two-week history of abdominal and right flank pain. He had a fever and was neutrophilic. On this occasion, an abscess on the right psoas muscle containing a calcified appendicolith was visible on CT (Figures 3 and 4).

**Figure 5:**
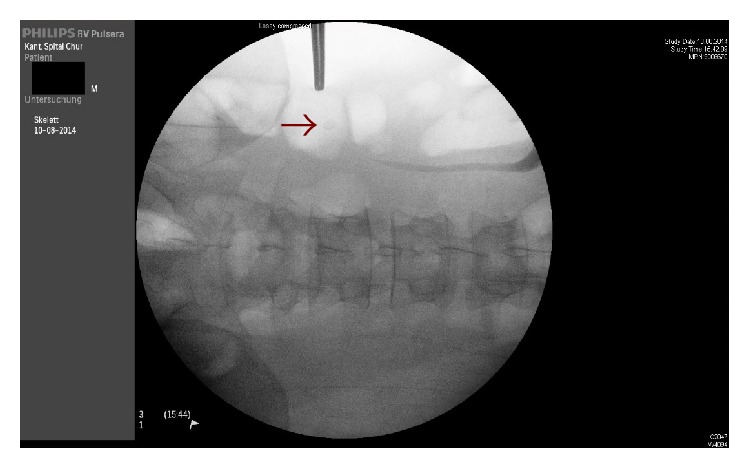


The abscess was drained via a right lumbotomy incision with the appendicolith identified under radiological guidance (Figure 5).

The appendicolith was palpated and extracted by finger. After two days, the patient was discharged in good health with antibiotics (amoxicillin/clavulanic acid) for seven days.

Two months later the patient was seen in Outpatient Clinic and eight months later he received a consultation by telephone. He reported no further pain and was healthy.

## 3. Discussion

This case demonstrates that an appendicolith can be highly mobile and change position over time. This can give rise to a spectrum of clinical signs and make diagnosis challenging, as demonstrated here. When the patient was referred with a suspected urinary calculus, the CT scan performed at that time actually revealed an abscess containing a stone, which was not identified at operation. In retrospect, the search and recovery of the stone should have been more aggressive, not least since it gave rise to another abscess a year later.

The presence of an appendicolith in acute appendicitis is well described [1–3]. One migratory example gave rise to a pleural empyema, which was managed by stone extraction [4]. Dabare described a different method of extraction of a pelvic appendicolith by ultrasound-guided single-port surgery [5]. Other surgical approaches for stone extraction, both transcutaneous and laparoscopic, are described [6, 7].

However, the ideal extraction method is ultimately dependent on the location of the stone and abscess. Here, we performed extraction by right lumbotomy. The abscess location may vary, with the perihepatic space being a common site [6, 8]. In our case, the first recurrent abscess was paracolic and the second was on the right psoas muscle. Due to this possibility of recurrent abscesses, there is value in rigorously ensuring that a stone is removed if suspected, including employing radiological techniques where necessary.

## 4. Conclusion

In conclusion, we suggest that any appendicolith diagnosed pre- or intraoperatively should be removed due to the risk of recurrent abscess formation. The intraoperative search for appendicoliths should be comprehensive and use ancillary techniques where necessary. In patients suffering from recurrent flank pain and abscesses after appendectomy, the possibility of a lost or overlooked appendicolith should be considered.

## References

[1] Ishiyama M., Yanase F., Taketa T. (2013). Significance of size and location of appendicoliths as exacerbating factor of acute appendicitis. *Emergency Radiology*.

[2] Salemis N. S. (2012). Giant appendicolith, a rare cause of chronic right iliac fossa pain. *American Surgeon*.

[3] Rollins M. D., Andolsek W., Scaife E. R. (2010). Prophylactic appendectomy: unnecessary in children with incidental appendicoliths detected by computed tomographic scan. *Journal of Pediatric Surgery*.

[4] Betancourt S. L., Palacio D., Bisset G. S. (2015). The ‘wandering appendicolith’. *Pediatric Radiology*.

[5] Dabare D., Higginson A., Toh S. (2014). A novel transcutaneous ultrasonography guided single port removal of a pelvic appendicolith. *Annals of the Royal College of Surgeons of England*.

[6] Maatouk M., Bunni J., Schuijtvlot M. (2011). Perihepatic abscess secondary to retained appendicolith: a rare complication managed laparoscopically. *Journal of Surgical Case Reports*.

[7] Hegarty C., Heaslip I., Murphy M., McDermott E. W., Brophy D. P. (2012). Percutaneous removal of a dropped appendicolith using a basket retrieval device and concomitant abscess drainage. *Journal of Vascular and Interventional Radiology*.

[8] Black M. T., Ha B. Y., Kang Y. S., Garland A. M. (2013). Perihepatic abscess caused by dropped appendicoliths following laparoscopic appendectomy: sonographic findings. *Journal of Clinical Ultrasound*.

